# Translation and cultural validation of the University of Washington Caregiver Stress and Benefit Scales

**DOI:** 10.1186/s41687-021-00394-y

**Published:** 2021-10-30

**Authors:** Dagmar Amtmann, Alyssa M. Bamer, Rana Salem, Arnold R. Gammaitoni, Bradley S. Galer, Mark P. Jensen

**Affiliations:** 1 Department of Rehabilitation Medicine, University of Washington, 12360 Lake City Way, Suite 502, Seattle, WA 98125 USA; 2 Medical and Scientific Affairs, Zogenix, Inc., 5858 Horton Street, Suite 455, Emeryville, CA 94608 USA; 3 Department of Rehabilitation Medicine, University of Washington, Box 359612, Seattle, WA 98195 USA

## Abstract

**Background:**

English versions of the University of Washington Caregiver Stress (UW-CSS) and Benefit (UW-CBS) Scales were developed in the United States (US) to measure impact on caregivers of caring for a child/children. Caregiving stress and benefit are important constructs to study worldwide. The purpose of this study was to translate and validate the UW-CSS and UW-CBS into French, German, Italian, and Spanish languages.

**Method:**

UW-CSS and UW-CBS were translated using forward and backward translation with reconciliation. Cognitive interviews (CIs) were completed with caregivers of children < 18 years with severe epilepsy. Translated versions were also administered to at least 100 caregivers in each of the four countries: France, Germany, Italy, and Spain. Differential item functioning (DIF) analyses were used to assess linguistic and cultural bias by country. The US development sample of 722 caregivers was used as a comparison sample for DIF analyses. DIF adjusted scores were calculated to determine impact of DIF on the item response theory (IRT)-based T-score. Benefit and stress scores were also calculated and compared across countries and health condition subgroups. Finally, short forms were modified to minimize the impact of DIF on the UW-CSS and UW-CBS T-scores and to reflect feedback from CIs.

**Results:**

Interviews were completed with 47 caregivers (German n = 14; Spanish n = 10; French n = 13; Italian n = 10). UW-CSS and UW-CBS were administered to 456 (German n = 117, Spanish n = 114, French n = 115, Italian n = 110) caregivers of children with and without health conditions. All stress items functioned well in CIs, though results indicated statistically significant DIF for three items in multiple countries and in the overall sample. Four of the 13 benefit items were problematic based on CI feedback, and six items showed DIF in one or more countries or in the combined sample. However, average differences between DIF adjusted and non-adjusted scores were minimal for both scales and all comparisons, indicating the impact of DIF on the total score was negligible.

**Conclusion:**

Modified short forms functioned well in all four of the translated versions. All language versions are freely publicly available.

## Background

Taking care of a child or children with chronic illness, such as severe epilepsy, can be stressful and exhausting for caregivers, but can also bring significant benefits and rewards. In order to better understand the impact of caregiving on caregivers, Amtmann et al. (2020) developed the University of Washington (UW) Caregiver Stress (UW-CSS) and Caregiver Benefit (UW-CBS) scales using item response theory (IRT) [1]. The UW-CSS and UW-CBS were developed in English for caregivers of healthy children or children with chronic conditions, and both scales demonstrated strong reliability and validity [1]. The measures were also designed to be relevant to caregivers of children with severe epilepsy, as caring for a child with severe epilepsy can have unique challenges. For both instruments, an IRT-based T-score of 50 represents the mean caregiver stress or benefit in United States (US) community sample of caregivers [1].

Due to differences in how societies support families and due to cultural differences, the stresses and benefits experienced by caregivers may differ across countries. In order to understand these differences, researchers need culturally and linguistically appropriate measures. The Functional Assessment of Chronic Illness Therapy (FACIT) is a rigorous translation methodology that includes forward and backward translation and pretest item review, and is recommended for translation of health outcome measures [2]. The objective of this study was to translate and validate the translations of the UW-CSS and UW-CBS into French, German, Italian, and Spanish languages utilizing FACIT translation methodology.

## Methods

### Participants and procedures

The UW-CSS and UW-CBS were translated into four languages using forward and backward translation with reconciliation. Semi-structured cognitive interviews (CIs) with caregivers were used to evaluate translated versions. Data collected in a large-scale administration were used to evaluate differential item functioning (DIF) and compare stress and benefit scores between European Union (EU) countries and the USA.

#### Translations

The UW-CSS and UW-CBS translations were conducted by The Academy of Languages Translation and Interpretation Services (AOLTI, https://aolti.com/) who specialize in medical translations. All language translations were back-translated to English by AOLTI. Trained native speakers of each language worked with the AOLTI to arrive at the final translations to be tested in CIs.

#### Cognitive interviews

Trained native speaker interviewers completed semi-structured CIs [3] over the phone or via web teleconference software (e.g., Zoom) with caregivers of children (< 18 years) with epileptic encephalopathies (EE). Interviews were recorded and the interviewers’ notes were used to compile summaries of feedback on each item. Caregivers were defined as a parent or legal guardian who coordinates and provides most of the unpaid day-to-day care for a child. Eligibility criteria included residing in France, Germany, Italy, or Spain, and ability to read, speak and understand French, German, Italian, or Spanish, respectively. Participants were recruited with help from clinicians who see patients with EE and from participants in previous studies [4]. A minimum of five caregivers reviewed each item in each language, with at least one male and two caregivers of younger children with EE (< 9 years). The interviews assessed the comprehension, clarity, and cultural applicability of the items. Items that required significant modifications after CI testing were tested in a second round of interviews with at least three participants. Two additional German caregivers of healthy children were recruited due to difficulties translating the term “caregiving” into German. Caregivers also completed a short online survey with demographic and clinical information. Surveys were administered through the REDCap (Research Electronic Data Capture) web-based software platform [5, 6]. Participants provided informed consent and were sent a €43 electronic gift card.

#### Large scale administration

The final translated and revised UW-CSS and UW-CBS items were administered to a larger sample (N = 400 target sample size) along with demographic and child health questions via an online survey also using REDCap [5, 6]. Adult caregivers (> 18 years) residing in France, Italy, Germany, or Spain and fluent in the native language of the country, and caring for at least one child under age 18 years were eligible. At least 100 caregivers per country was targeted, with additional subsample targets per country: 50 caregivers of a child with EE, 25 caregivers of a child with a chronic health condition, and 25 caregivers of children with no health conditions. Caregivers were recruited from the CI study and by Op4G (https://op4g.com/), a market research organization. Participants recruited by Op4g were not paid but participants recruited from the CI study were sent an €23 electronic gift card after completing the survey.

### Analyses

#### Cognitive interviews

Any problematic or confusing items were flagged and addressed. Minor changes were made to the English version to keep content and constructs as consistent as possible across all versions.

#### Differential item function

DIF analyses were conducted using data from the large-scale administration to examine the linguistic and cultural equivalence of the translations. The original US development sample, described in detail in Amtmann et al. [1] was used as a comparison for the analyses. The US development sample included a mix of caregivers of children with EE, Down syndrome, muscular dystrophy, or children with no specific health care needs. Prior to running DIF analyses unidimensionality of the scales was examined using 1-factor confirmatory factor analysis (CFA) using Mplus software 8.2. [7]. A comparative fit index (CFI) of 0.90 or higher was considered sufficient support for unidimensionality [8]. DIF was assessed by each country individually (e.g., US vs Spain) as well as by the combined sample (i.e., US vs EU) using the program *lordif* [9] in R [10] with an *R*^*2*^ criterion of 0.02, as is recommended for translation validity analyses [11]. If statistically significant DIF was observed DIF adjusted scores were calculated and compared to non-adjusted scores to determine the scale-level impact of DIF [12].

#### US and EU comparisons

Sample demographics were compared using Student’s t-tests or chi-squared tests. UW-CSS and UW-CBS 6-item short form scores were generated and summarized across countries and subgroups. Using the Student’s t-test, stress and benefit scores in EU countries were also compared to scores in the US sample utilized for the DIF analyses.

#### Short Forms

Fixed length short forms developed by Amtmann et al. [1] were revised based on the results of the CIs and DIF analyses. Items that were identified as problematic were removed and\or replaced with better functioning items and items without DIF. Internal consistency of the new short forms was examined using Chronbach’s alpha [13] and item convergent validity by calculating corrected item-total score correlations. Alpha values between 0.7 and 0.9 and correlations > 0.40 were considered acceptable [14].

## Results

### Cognitive interview study

Interviews were completed with 47 parent caregivers (German n = 14; Spanish n = 10; French n = 13; Italian n = 10) (see Table 1 for sample demographics). All but two participants cared for a child with EE.

**Table 1:** Demographics of study samples in the European Union and the United States

	EU Cognitive Interview Sample	EU Large Scale Administration Sample	US Development Sample	Comparison test for EU Large Scale with US Development Sample t-test t, p
	N = 47	N = 456	N = 722	
	Mean (SD) [Range]	Mean (SD) [Range]	Mean (SD) [Range]	
Caregiver age (years)	41.3 (6.5) [31.9 – 65.1]	39.6 (6.1) [20.9 – 65.7]	41.5 (8.5) [20.0 – 72.0]	4.15, p < 0.001
Child/children’s age (years)	8.1 (3.9) [2.3 – 16.7]	9.7 (3.7) [0.7 – 17.9]	9.1 (4.9) [0.1 – 17.9]	2.57, p = 0.01

	n (%)	n (%)	n (%)	Chi^2^, *p*
*Caregiver gender*				0.59, p = 0.44
Female	36 (77%)	369 (81%)	597 (83%)	
Male	11 (23%)	87 (19%)	125 (17%)	
*Country*				
France	13 (28%)	115 (25%)	–	
Germany	14 (30%)	117 (26%)	–	
Italy	10 (21%)	110 (24%)	–	
Spain	10 (21%)	114 (25%)	–	
*Caregiver education*				50.6, p < 0.001
Some HS or less	8 (17%)	17 (4%)	22 (3%)	
HS graduate/GCSE	17 (36%)	40 (9%)	102 (14%)	
Some university/vocational	8 (17%)	185 (41%)	208 (29%)	
University degree	5 (11%)	171 (38%)	224 (31%)	
Advanced degree	8 (17%)	43 (9%)	165 (23%)	
*Caregiver employment status*				2.82, p = 0.09
Employed	24 (51%)	299 (66%)	439 (61%)	
Unemployed, Homemaker, Retired	22 (47%)	154 (34%)	279 (39%)	
*Child's health status*				179.8, p < 0.001
Epilepsy	45 (96%)	251 (55%)	128 (18%)	
Other chronic health condition	0 (0%)	105 (23%)	272 (38%)	
Healthy/community sample*	2 (4%)	100 (22%)	322 (45%)	

*EU sample excluded families with children with serious chronic conditions while the US sample was a community-based sample without exclusions for chronic conditions

Based on CI feedback the instructions for both scales were modified in all languages (including English) to clarify that “caregiving” refers to “all aspects” of taking care of a child or children and to take into account how “having a child or children you take care of affects all areas of your life.” Because the instructions define caregiving as “typically unpaid,” in the German translation we added a statement that the government stipend paid to German parents and guardians to help with caregiving (“*Pflegegeld*”) did not count as paid caregiving when responding to the questions.

The UW-CSS translated items functioned well, although some translations required minor changes to improve comprehension and to clarify meaning. CIs identified issues with three UW-CBS items that did not work well in all languages (being a better advocate, putting life in perspective and feeling closer to other adults) and a fourth item (being more accepting) was problematic in German (Table 2). Concepts in these items were both difficult to translate into other languages, and the translated items were difficult for caregivers to understand. Short forms were modified to exclude these problematic items. Several caregivers also felt that the benefit items were repetitive (for example, the benefits of “finding new strengths” and “becoming a stronger person”).

**Table 2:** Summary of cognitive interview and differential item functioning results for the final translated UW-CSS and UW-CBS items

	Revised/final item English text	CI results summary	DIF compared to US sample
*UW caregiver stress items*			
care01	How much are your finances strained because of caregiving?		
care04	How much does your own health suffer because of caregiving?		
care11	How difficult is it for you to get a good night's sleep because of caregiving?*		
care17	How difficult is it for you to take care of your household because of caregiving?		
care83	How difficult is it to find time to spend with your friends because of caregiving?*		
care84	How much do you feel always "on call" because of caregiving?		EU, Fr, Sp, It
care87	How difficult is it for you to take care of yourself because of caregiving?**		
care36	How difficult is it to make plans because of caregiving?		
care37	How difficult is it to do activities you like to do because of caregiving?**		
care88	How difficult is it to do things that are important to you because of caregiving?		
care43	How much does caregiving limit your work opportunities?		
care29	On a typical day, how often do you feel overwhelmed by caregiving?**		
care34	How often do you feel socially isolated because of caregiving? *		
care38	How often do caregiving responsibilities make you feel physically exhausted?		
care39	How often do caregiving responsibilities make you feel mentally exhausted?		EU, Ge, Fr, It
care12	How often does caregiving make it difficult for you to take care of your health?		
care44	How often do you need to miss work because of caregiving?		
care49	How often is it difficult to do things you enjoy with your partner because of caregiving?		EU, Ge, Fr
care45	How much does caregiving strain your relationship with your partner?		
*UW caregiver benefit items*			
care02	How much does caregiving help you appreciate what is important in life?		EU, Fr, Sp, It
care07	How much does caregiving help you find new strengths in yourself?*		
care10	Are you a better advocate for your child/children because of caregiving?	Ge, Fr, Sp, It	
care19	How much do you feel caregiving has made you a better person?*		
care20	How much do you feel that caregiving has helped you put life in perspective?	Ge, Fr, Sp, It	EU, Fr
care24	How much does caregiving help you become a more patient person?*		
care30	How much do you feel that caregiving has made you a stronger person?**		
care08	Have you gained confidence in yourself because of caregiving?		EU, Ge
care13	How much does caregiving add meaning to your life?**		Fr
care15	How much does caregiving make you a more accepting person?	Ge	
care23	How much does caregiving help you be more caring?**		
care26	How often does caregiving make you feel closer to other adults who are important to you?	Ge, Fr, Sp, It	EU, Ge, Sp, It
care48	How often do you feel closer to your partner because of caregiving?		EU

US, United States; EU, European Union countries combined; Ge, Germany; Sp, Spain; Fr, France; It, Italy

*Item is included on the recommended 6-item short form

**Item is included on both the recommended 3- and 6-item short forms

An additional issue relating to translation of “caregiving” in German was also identified, as there are two terms for caregiving. *Erziehung* and *Betreuung* are more commonly used and describe the process to raise and educate (*Erziehung*) and care and support (*Betreuung*) a child, respectively. *Fürsorge* is used to describe caring for a child with a chronic health condition. In addition to the 12 German caregivers of children with EE, two German participants who cared for healthy children with no chronic health conditions were interviewed to get their thoughts on the best word or words to describe “caregiving” in German. Feedback from the 12 German caregivers of children with EE and two who cared for healthy children indicated that combining the two terms into one (i.e., “*Erziehung/Betreuung”*) would be acceptable.

### Large scale administration study

A total of 456 caregivers from France (n = 115), Germany (n = 117), Italy (n = 110), and Spain (n = 114) completed the study (see Table 1 for sample demographics).

#### Differential item function

Unidimensionality of both scales was supported by 1-factor CFA, with CFI values of 0.90 for stress and 0.98 for benefit. DIF analyses identified three stress items with statistically significant DIF by multiple countries and in the overall sample (see Table 2). Similarly, six benefit items displayed DIF in one or more countries or in the combined sample. However, average differences between DIF adjusted and non-adjusted scores were less than 1 point on the T-score metric, for both scales and for all comparisons.

#### US and EU score comparisons

Sample demographic differences are shown in Table 1. The epilepsy and community subsamples in the EU reported less stress than corresponding US subsamples (both *p’s* < 0.001) (see Table 3). The overall EU sample and community subsamples also reported less benefit than parents in the US (both *p*’s < 0.001). Additional comparisons between samples and subgroups are shown in Table 3.

**Table 3:** Caregiver stress and benefit scores and comparisons across subgroups and countries in the European Union and the United States

	Sample size		Stress			Benefit		
	US	EU	US	EU	T-test comparison	US	EU	T-test comparison
	n		Mean (SD)	Mean (SD)	t, p-value	Mean (SD)	Mean (SD)	t, p-value
Full sample	717	455	54.1 (9.3)	53.4 (7.2)	1.4, 0.16	47.9 (9.2)	44.5 (8.1)	6.3, < 0.001
Epilepsy	128	251	62.8 (6.6)	55.8 (5.1)	11.3, < 0.001	45.8 (9.0)	44.3 (7.6)	1.7, 0.08
Chronic health condition	272	105	54.4 (8.2)	54.4 (5.3)	0.004, 0.99	46.8 (8.8)	47.4 (7.3)	0.7, 0.5
Community/healthy sample*	321	99	50.5 (8.8)	46.3 (9.0)	4.1, < 0.001	49.6 (9.4)	42.1 (9.2)	7.1, < 0.001
France		115		52.3 (7.9)			46.2 (8.4)	
Italy		110		54.0 (6.9)			43.7 (7.9)	
Germany		117		53.3 (6.3)			43.8 (7.9)	
Spain		114		54.2 (7.7)			44.3 (8.0)	

*EU sample excluded families with children with serious chronic conditions while the US sample was a community-based sample without exclusions for chronic conditions

#### Short forms

Previously published short forms were modified by excluding problematic items to minimize the impact of DIF on the UW-CSS and UW-CBS T-scores and to reflect feedback from CIs. New 6-item and 3-item short forms are recommended as indicated in Table 2. Correlations between the 6-item and 3-item UW-CSS short forms scores and the full bank were 0.96 and 0.94, respectively. Similarly, correlations for UW-CBS were 0.97 and 0.92. Cronbach’s alpha values for the 6-item short forms were 0.83 for stress and 0.85 for benefit. Corrected item-total correlations ranged from 0.52 to 0.66 for stress and 0.56 to 0.74 for benefit.

## Conclusions

The UW-CSS and UW-CBS were translated into French, Italian, German, and Spanish using rigorous methods. CI feedback resulted in minor changes to the English version to improve functioning of items and to harmonize items across all languages. CIs also identified benefit items that were difficult to translate. The items, “put life into perspective” and “be a better advocate,” were both difficult to translate into each of the four languages and hard to understand conceptually; caregivers often said they did not see how these concepts were related to caregiving. For the item “feel closer to other adults who are important to you,” caregivers did not know who this meant—their partner, parents, and whether this meant spending time with these people or feeling connected to them. This feedback indicated important cultural differences between US and EU countries, and perhaps explains why EU samples generally report less caregiver benefit. To address this difference, new short forms were made that prioritized items that did not show bias by country, to minimize any potential DIF impact. All language versions of short forms for the UW-CSS and UW-CBS are free and publicly available including user guides with scoring [15]. The 6-item short forms are recommended in most situations. The 3-item short form minimizes respondent burden and is appropriate for group level comparisons.

The primary limitation of this study is related to the power to detect DIF by country and relatively small sample size of the caregivers in the specific categories in each country. The EU sample sizes for DIF by country were below that recommended to detect DIF [16], and additional DIF may exist that we may not have detected due to sample size limitations. We also note that the DIF detected may be partially related to differences in education and child health status between the samples and not just reflective of linguistic differences, though no DIF was detected by education or child diagnosis in the original validation study [1]. An additional limitation of this study is that it only included a limited number of health conditions and cognitive interviews were primarily conducted with caregivers of children with Epilepsy. Future studies should be conducted to further validate the measures in other health conditions not included in this study and could be done to further explore the responsiveness of the scales and the nature of the differences between caregiver benefits between the US and EU caregivers.

This study developed translations of UW-CSS and UW-CBS in four EU languages. New English, Spanish, Italian, French and German short forms were also created. The scales can be used in research and clinical practice to examine caregiver stress and benefit experienced by caregivers of healthy children, as well as caregivers of children with health conditions, including severe epilepsy.

## Data Availability

The datasets analyzed during the current study are available from dagmara@uw.edu on reasonable request.

## Abbreviations

UW-CSS: University of Washington Caregiver Stress Scale
UW-CBS: University of Washington Caregiver Benefit Scale
US: United States
CI: Cognitive Interview
DIF: Differential Item Functioning
IRT: Item Response Theory
FACIT: Functional Assessment of Chronic Illness Therapy
EU: European Union
AOLTI: Academy of Languages Translation and Interpretation Services
EE: Epileptic encephalopathies
SD: Standard deviation
